# MET18 Connects the Cytosolic Iron-Sulfur Cluster Assembly Pathway to Active DNA Demethylation in *Arabidopsis*

**DOI:** 10.1371/journal.pgen.1005559

**Published:** 2015-10-22

**Authors:** Cheng-Guo Duan, Xingang Wang, Kai Tang, Huiming Zhang, Satendra K. Mangrauthia, Mingguang Lei, Chuan-Chih Hsu, Yueh-Ju Hou, Chunguo Wang, Yan Li, W. Andy Tao, Jian-Kang Zhu

**Affiliations:** 1 Department of Horticulture and Landscape Architecture, Purdue University, West Lafayette, Indiana, United States of America; 2 Shanghai Center for Plant Stress Biology, Shanghai Institute of Biological Sciences, Chinese Academy of Sciences, China; 3 Biotechnology Section, Indian Institute of Rice Research (IIRR), Hyderabad, India; 4 Department of Biochemistry, Purdue University, West Lafayette, Indiana, United States of America; 5 College of Life Sciences Nankai University, Tianjin, China; 6 Ecological Security and Protection Key laboratory of Sichuan Province, Mianyang Normal University, Mianyang, Sichuan, China; National Institute of Genetics, JAPAN

## Abstract

DNA demethylation mediated by the DNA glycosylase ROS1 helps determine genomic DNA methylation patterns and protects active genes from being silenced. However, little is known about the mechanism of regulation of ROS1 enzymatic activity. Using a forward genetic screen, we identified an anti-silencing (ASI) factor, ASI3, the dysfunction of which causes transgene promoter hyper-methylation and silencing. Map-based cloning identified ASI3 as MET18, a component of the cytosolic iron-sulfur cluster assembly (CIA) pathway. Mutation in *MET18* leads to hyper-methylation at thousands of genomic loci, the majority of which overlap with hypermethylated loci identified in *ros1* and *ros1dml2dml3* mutants. Affinity purification followed by mass spectrometry indicated that ROS1 physically associates with MET18 and other CIA components. Yeast two-hybrid and split luciferase assays showed that ROS1 can directly interact with MET18 and another CIA component, AE7. Site-directed mutagenesis of ROS1 indicated that the conserved iron-sulfur motif is indispensable for ROS1 enzymatic activity. Our results suggest that ROS1-mediated active DNA demethylation requires MET18-dependent transfer of the iron-sulfur cluster, highlighting an important role of the CIA pathway in epigenetic regulation.

## Author Summary

DNA cytosine methylation is a major epigenetic mark that confers transcriptional regulation. Active removal of DNA methylation is important for plants and mammals during development and in responses to various stress conditions. In the model plant species *Arabidopsis thaliana*, active DNA demethylation depends on a family of 5-methylcytosine DNA glycosylases/demethylases including ROS1, DME, and others. While the epigenetic function of this demethylase family is well-known, little is known about how their enzymatic activities may be regulated. In this report, we carried out a forward genetic screen for anti-silencing factors and identified MET18, a conserved component of cytosolic iron-sulfur cluster assembly (CIA) pathway in eukaryotes, as being required for the ROS1-dependent active DNA demethylation. Dysfunction of MET18 causes DNA hyper-methylation at thousands of genomic loci where DNA methylation is pruned by ROS1. In addition, ROS1 physically interacts with MET18 and other CIA pathway components; while a conserved iron-sulfur-binding motif is indispensable for ROS1 enzyme activity. Our results suggested that MET18 affects DNA demethylation by influencing ROS1 enzymatic activity via direct interaction with the iron-sulfur-binding motif of ROS1, highlighting a direct connection between iron-sulfur cluster assembly and active DNA demethylation.

## Introduction

DNA 5-cytosine methylation (5mC) is an epigenetic mark that is critical for maintaining genome integrity, regulating gene expression and responding to environmental stress in many higher eukaryotes [1–6]. A proper DNA methylation pattern is important for plant development and plant responses to various stress conditions [1,7]. In *Arabidopsis*, the DNA methylation pattern is established and maintained through different pathways by several DNA methyltransferases such as MET1, CMT3, DRM2 and CMT2 [1,8]. Active removal of 5mC also contributes to the steady state of DNA methylation levels [9]. Defects in active DNA demethylation can result in abnormal gene silencing due to increased DNA methylation in gene regulatory sequences [10,11].

In *Arabidopsis*, the ROS1/DME family of DNA glycosylases/lyases initiates active DNA demethylation by excising the 5mC and then cutting the phophodiester backbone through either β- or β, δ-elimination [2,6,10,12–14]. The β, δ-elimination generates a 3’ phosphate at the gap, which is then hydrolyzed by phosphatase ZDP [15], while β-elimination generates a 3’-phosphor-α, β- unsaturated aldehyde (3’-PUA), which is processed to a hydroxyl group by APE1L [16]. DNA polymerase and ligase fill the gap with an unmethylated cytosine through the base-excision repair pathway [2]. DNA demethylation by DME in the central cell contributes to parental specific expression of some imprinted genes in the endosperm, whereas ROS1 and other family members mainly affect the DNA methylation status in vegetative tissues [2]. The recruitment of ROS1 to the chromatin requires histone acetylation, which is catalyzed by IDM1 [11]. The *in vivo* activity of IDM1 and its targeting require other anti-silencing factors such as IDM2, IDM3 and MBD7 [17,18]. In addition, ROS3, which is an RNA-binding protein, has been suggested to mediate ROS1 recruitment at some loci because its mutation disrupts the subnuclear localization pattern of ROS1 and increases DNA methylation [19]. It is unclear, however, how the enzymatic activity of ROS1 may be regulated.

Many DNA-repair and -replication proteins contain iron-sulfur (Fe-S) clusters as cofactors [20,21], and such clusters are important for the enzymatic function and/or stability of the proteins. ROS1/DME DNA glycosylases are predicted Fe-S cluster-binding proteins since they have a conserved Fe-S-binding motif. A previous study has shown that the predicted Fe-S-binding motif is essential for DME enzymatic activity [22]. Eukaryotes have the ISC (iron–sulfur cluster assembly) apparatus in mitochondria, the SUF (sulfur mobilization) pathway in mitochondria and the CIA (cytosolic Fe-S assembly) pathway in cytoplasm for the assembly of Fe-S proteins in respective cellular compartments [23,24]. The CIA pathway is mainly responsible for the maturation of cytosolic and nuclear Fe-S proteins and depends on parts of the ISC pathway for a sulfur-containing component [23,24]. In the CIA pathway, two P loop NTPases, Cfd1 and Nbp35, form a protein complex which serves as a scaffold for Fe-S cluster assembly [25,26]. The Fe-S cluster is assembled to its binding protein by the CIA targeting complex, which consists of NAR1, CIA1, AE7, and MET18 in *Arabidopsis* [27]. In the complex, NAR1 shares sequence homology with Fe hydrogenases and binds to the Fe-S cluster [28]. CIA1 is a WD40 protein that functions as a scaffold for protein interactions, and AE7 is a DUF59 family protein [27,29]. MET18 is an ARM repeat-containing protein, which is a conserved component in the CIA pathway from yeast to plant to animal [21,23]. In yeast and animal cells, MET18 is the delivering protein that directly interacts with various Fe-S cluster-binding proteins [21,30,31].

Here, we identified MET18 as a component required for preventing transgene silencing as well as for normal DNA methylation patterns in *Arabidopsis*. We show that mutations in *MET18* result in genome-wide DNA hypermethylation patterns similar to those in the *ros1* mutant. We found a direct interaction between MET18 and ROS1, which suggests that MET18 is likely the end of the CIA pathway that transfers the Fe-S cluster to its apoprotein. These results also reveal for the first time a direct connection between the CIA pathway and DNA demethylation, and thus make a significant contribution to understanding how iron-sulfur cluster assembly affects epigenetic regulation.

## Results

### ASI3 is an anti-silencing factor

**Fig 1:**
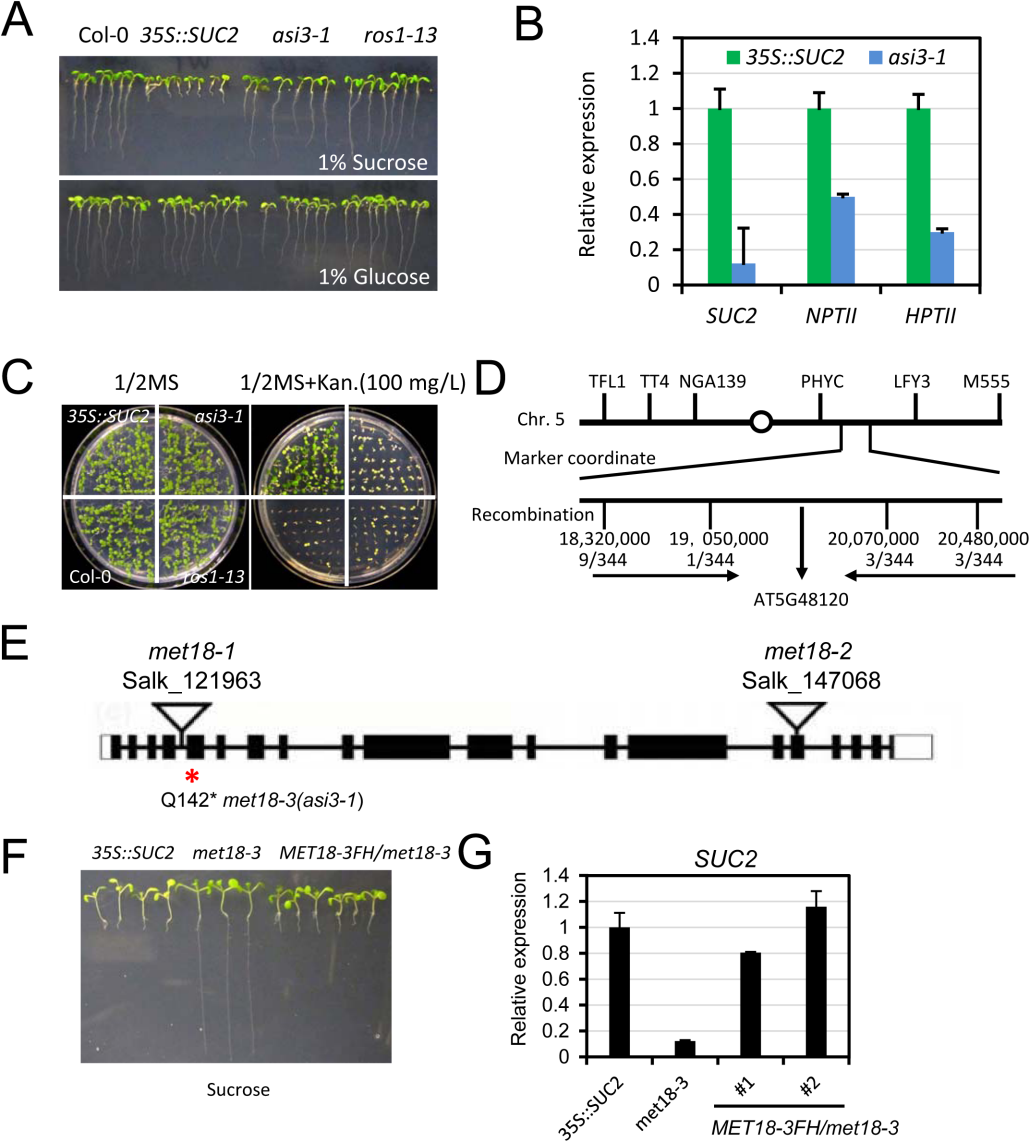
Characterization of the *asi3-1* (*met18-3*) mutant. A. Root phenotypes of the *asi3-1* mutant on ½ MS medium containing 1% glucose or sucrose. Seedlings were photographed 14 days after germination. Col-0, *35S*::*SUC2*, and *ros1-13* were used as controls. B. Quantitative RT-PCR results showing the relative expression levels of *SUC2*, *NPTII*, and *HPTII* transgenes in *asi3-1* mutant and *35S*::*SUC2* seedlings. *ACTIN 2* was used as an internal control. C. Kanamycin-sensitivity assay. D. Map-based cloning of the *asi3-1* mutation. E. The gene model structure of the *AT5G48120* (*ASI3/MET18*) gene. A Q-to-stop codon mutation in the 142nd amino acid of the *AT5G48120* gene is in *asi3-1* (*met18-3*) mutant. Two other T-DNA insertion mutants are shown. The exon, intron, and UTR regions are marked by a black box, black line, and white box, respectively. F. Root phenotypes of the *MET18-3FH*/*asi3-1* transgenic lines. Several randomly selected transgenic plants in the T3 generation were grown on ½ MS medium containing 1% sucrose. Seedlings were photographed 12 days after germination. G. Quantitative RT-PCR results showing the *SUC2* transgene expression levels in *MET18-3FH* transgenic plants.

To search for anti-silencing factors in *Arabidopsis*, we previously developed a forward genetic screen system in *Arabidopsis* (Wang et al., 2013; Lei et al., 2014). In this system, the *SUCROSE TRANSPORTER 2* (*SUC2*) gene driven by the cauliflower mosaic virus 35S promoter is overexpressed in Col-0 transgenic plants (referred to as *35S*::*SUC2*). When grown on a medium containing sucrose, *35S*::*SUC2* seedlings produce short roots because the roots over-accumulate sucrose (Fig 1A). Both *35S*::*SUC2* and mutant plants grow normally on glucose-containing medium (Fig 1A). We generated an ethylmethane sulfonate (EMS)-mutagenized population and screened for mutants with long-root phenotype on sucrose-containing medium. With this strategy, many known components associated with DNA methylation were identified, including the loss-of-function of DNA demethylase ROS1 mutants (Fig 1A) and also RNA-directed DNA methylation (RdDM) mutants [32–34]. In this study, we identified a new recessive mutant named *anti-silencing 3–1* (*asi3-1*) with a long-root phenotype (Fig 1A). We first examined the expression of the *SUC2* transgene by real-time quantitative RT-PCR (qRT-PCR). Compared to its expression in *35S*::*SUC2* plants, *SUC2* expression in *asi3-1* plants was significantly reduced (Fig 1B). *35S*::*SUC2* plants also express two marker genes, *neomycin phosphotransferase II* (*NPTII*) and h*ygromycin phosphotransferase II* (*HPTII*), driven by shorter forms of 35S promoter for transgenic plant selection. In contrast to *35S*::*SUC2* plants, which grew well on a kanamycin-containing medium, *asi3-1* and *ros1-13* were sensitive to kanamycin (Fig 1C). qPCR results indicated that the sensitivity was due to a reduction in *NPTII* transgene expression (Fig 1B). Moreover, *HPTII* expression was also down-regulated in the *asi3-1* mutant (Fig 1B). The observation that ASI3 dysfunction leads to down-regulation of transgenes suggested that ASI3 may function as an anti-silencing factor that prevents transgene silencing in *Arabidopsis*. In addition to transgene silencing, the *asi3-1* mutant also displayed morphological phenotypes such as reduced plant and leaf sizes (S1A Fig).

Through genetic mapping, the *asi3-1* mutation was mapped to a narrow region between two simple sequence length polymorphism (SSLP) markers located on chromosome 5: 19,050,000 and 20,070,000 (Fig 1D). By whole genome re-sequencing, we detected in this region a “CAA” to “TAA” mutation in the first exon of *AT5G48120* gene, which encodes the Armadillo repeat motif (ARM)-containing protein MET18. This mutation causes an amino acid change from Q to a stop codon, likely leading to premature termination of translation (Fig 1E). To further confirm that the mutation in *AT5G48120* caused the silencing phenotype, we cloned the wild-type genomic sequence of *AT5G48120* and transformed it into the *asi3-1* mutant. Like the *35S*::*SUC2*, the transgenic plants exhibited a short-root phenotype (Fig 1F). We selected the transgenic lines with resistance to Basta and assessed the expression of the recombinant protein MET18-3FLAG-3HA (MET18-3FH) in the T1 generation by Western blotting (S1B Fig). The *SUC2* transgene expression was recovered to the *35S*::*SUC2* level, indicating that the *AT5G48120* gene complemented the transgene-silencing phenotype of the *asi3-1* mutant (Fig 1G). Moreover, the reduced-plant size phenotype was rescued by the introduction of *MET18* genomic DNA, indicating that this developmental defect was caused by MET18 dysfunction (S1B Fig). All of these results confirmed that A*T5G48120* is the gene corresponding to the *asi3-1* mutation. *AT5G48120* (*MET18*) encodes an ARM repeat superfamily protein, which was previously identified as a homolog of yeast Met18 [27]. Because two T-DNA insertion alleles (*met18-1* and *met18-2*) have been previously identified (Fig 1E) [27], we named the *asi3-1* mutant *met18-3* (Fig 1E).

### MET18 dysfunction causes genome wide DNA hypermethylation

**Fig 2:**
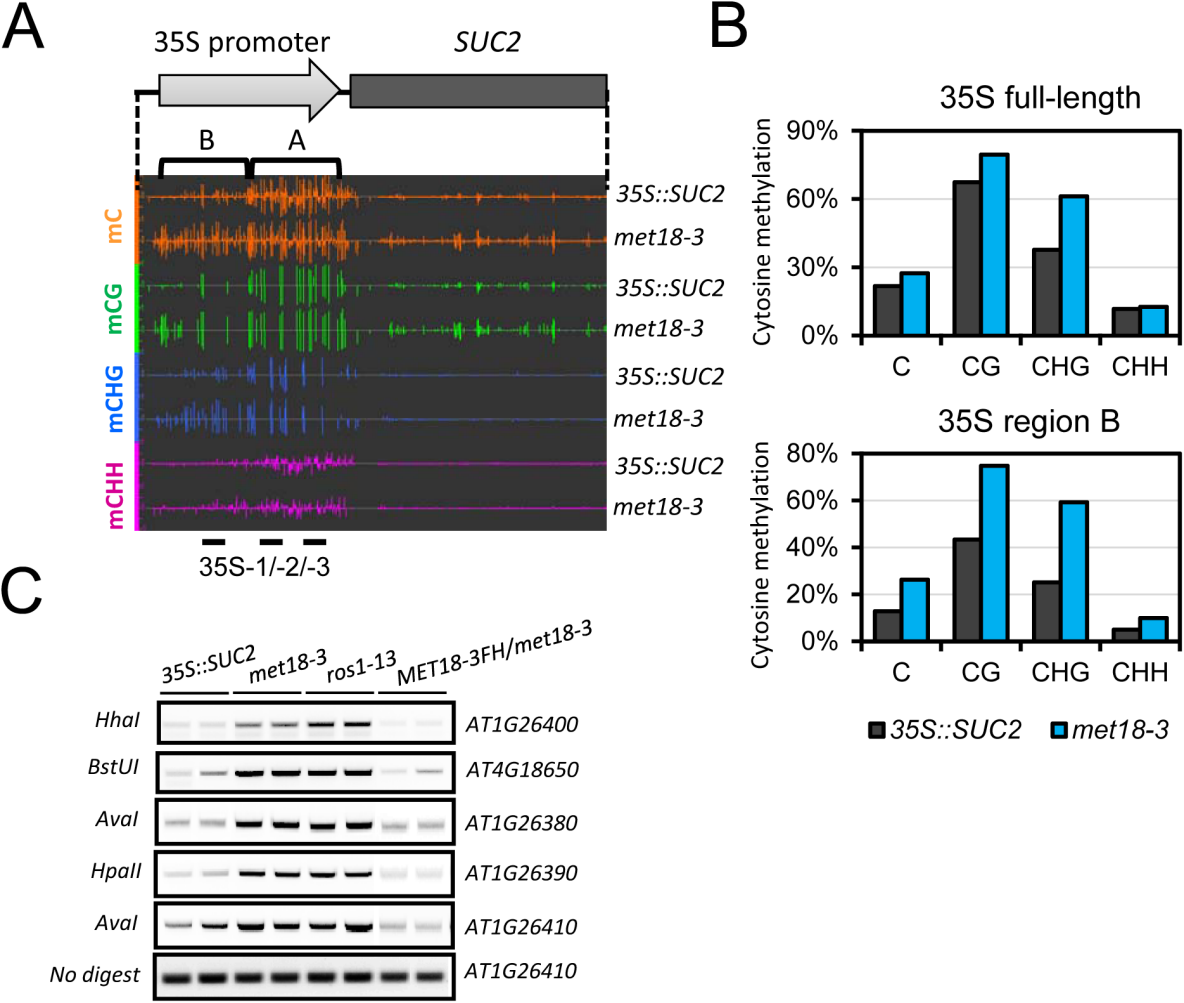
Epigenetic modification changes of the *35S*::*SUC2 transgene* promoter in *met18-3* mutant. A. Diagram of the *35S*::*SUC2* transgene and an IGB snapshot of DNA methylation levels in the promoter region in different cytosine contexts. The positions of primer pairs used in ChIP-qPCR are as labeled. B. DNA methylation levels in the promoter region of *35S*::*SUC2*. DNA methylation levels were quantified based on whole-genome bisulfite sequencing data. C. DNA methylation-sensitive PCR (Chop-PCR) results showing increased DNA methylation in *met18-3* and *ros1-13* mutants. Complementation with *MET18* genomic sequence rescued increased DNA methylation. Non-digested DNA was used as the control PCR template.

To investigate whether silencing of transgenes was associated with DNA methylation, we performed whole-genome bisulfite sequencing (WGBS) of *35S*::*SUC2* and *met18-3* mutant seedlings. We examined the DNA methylation level in the *35S*::*SUC2* promoter region, which carries ~800 bp full-length 35S promoter different from the 35S promoters of *NPTII* and *HPTII* transgenes (~400bp containing two tandem repeats of 35S 3’ terminal sequence) (Fig 2A). The whole-genome methylation data showed clear increases in methylation in all cytosine contexts (CG, CHG, and CHH) in the *met18-3* mutant compared to the *35S*::*SUC2*, and especially in the upstream region (Region B) of the 35S promoter (Fig 2A and 2B). These results indicated that *SUC2* transgene silencing was associated with DNA hypermethylation-mediated transcriptional repression. To confirm the DNA hypermethylation phenotype in *met18-3*, DNA methylation-sensitive PCR (Chop PCR) was performed. The results indicated that *met18-3* displays a hypermethylation phenotype in all five examined loci (Fig 2C) that were previously identified as ROS1-target loci [11,33,34]. As a positive control, *ros1-13* also showed hypermethylation at the examined loci (Fig 2C). As expected, introduction of *MET18* genomic DNA rescued the DNA methylation phenotype in the *met18-3* mutant, indicating that MET18 dysfunction is responsible for the hypermethylation phenotype in *met18-3*. Moreover, unlike RdDM mutants in which the transcript level of *ROS1* gene is reduced [33,34], MET18 dysfunction did not affect *ROS1* mRNA level (S2 Fig), suggesting that MET18 may be a new factor critical for DNA demethylation.

**Fig 3:**
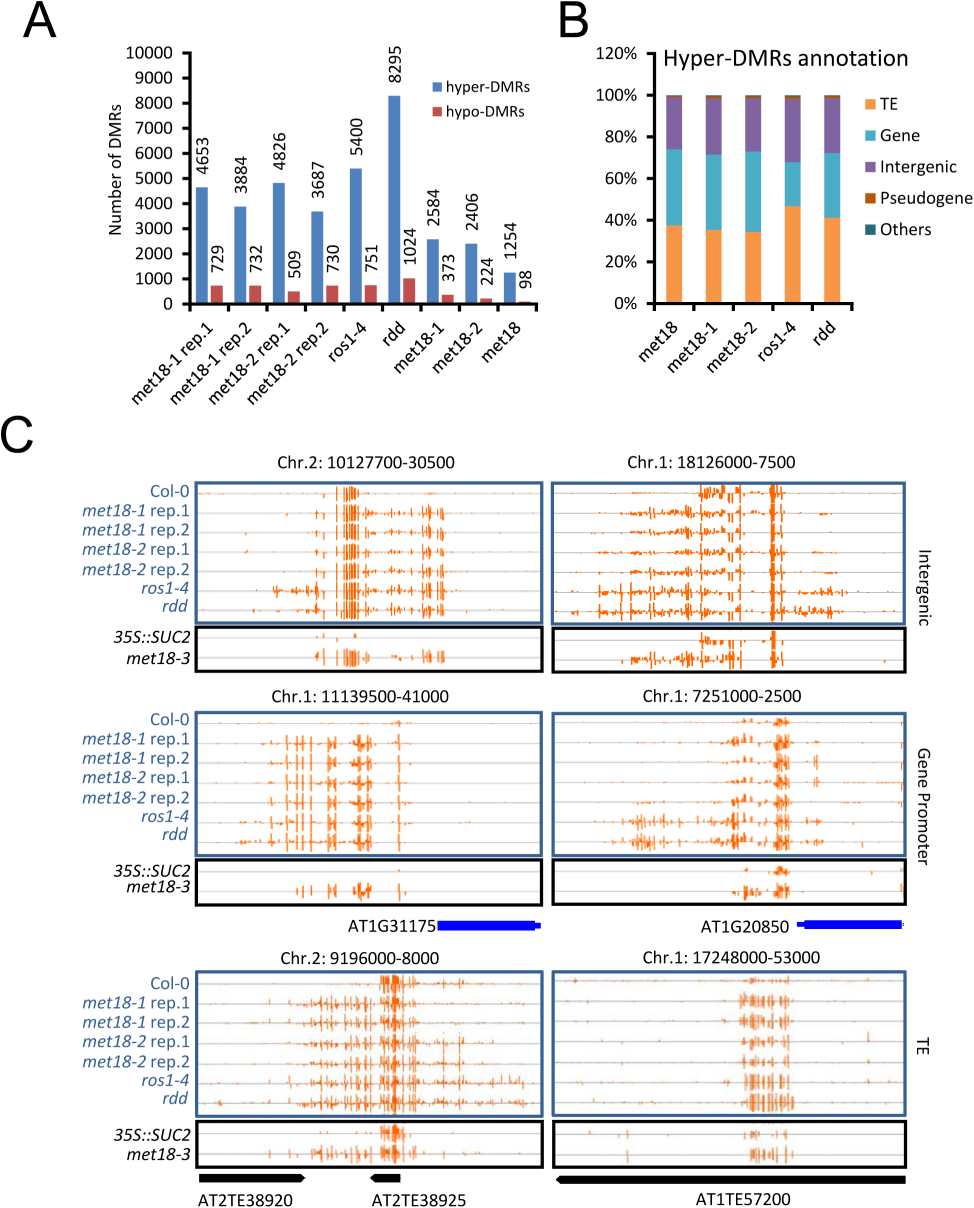
DNA methylation profiles in the *met18*, *ros1-4* and *rdd* mutants. A. Numbers of hyper- and hypo-DMRs identified in each mutant. *met18-1* rep.1 and *met18-1* rep.2 are two replications of *met18-1*; *met18-2* rep.1 and *met18-2* rep.2 are two replications of *met18-2*. *met18-1* represents the overlap of two *met18-1* replications and *met18-2* represents the overlap of two *met18-2* replications. *met18* represents the overlap of all four samples of *met18* mutants. B. Compositions of hyper-DMRs identified in different mutants. TE, DMRs in transposons but not in protein-coding genes; Gene, DMRs in protein-coding genes but not in TEs; IG, intergenic regions; Other, DMRs that overlap with other features that do not belong to TE, Gene, or IG regions. C. Snapshots of some representative DNA hypermethylation loci in *met18*, *ros1-4*, and *rdd* mutants. Hyper-DMRs in the intergenic regions (upper panel), gene promoter regions (middle panel), and TE regions (lower panel) are shown. Blue and black words indicate plants in Col-0 and *35S*::*SUC2* transgene background, respectively. The exact length and chromosome coordinates of the hyper-DMRs are listed in the S1 Table.

To further investigate the effects of MET18 dysfunction on whole genome DNA methylation level, we subjected two T-DNA insertion alleles, *met18-1* and *met18-2* [35](Fig 1E), for whole-genome bisulfite sequencing. Two biological replicates were sequenced for each allele (S3 Fig). Using the published Col-0 WGBS data as the control [11], we identified thousands of differentially methylated regions (DMRs) in the mutants. There are 4653/729, 3884/732, 4826/509 and 3687/730 hyper/hypo-DMRs in *met18-1* replicate 1(rep.1), *met18-1* rep.2, *met18-2* rep.1 and *met18-2* rep.2, respectively (S1 Table and Fig 3A and 3C). The predominance of hyper-DMRs in all 4 WGBS datasets strongly supports our hypothesis that MET18 has a negative effect on DNA methylation. We then compared the DMRs between different replicates and different mutant alleles. There are 2584 and 2406 overlapping hyper-DMRs between two technical replicates of *met18-1* and *met18-2*, respectively (S4 Fig). The percentages of overlapping hyper-DMRs between replicates are about 56% (*met18-1* rep.1) / 67% (*met18-1* rep.2) and 50% (*met18-2* rep.1) / 65% (*met18-2* rep.2) (S4 Fig and S1 Table), respectively, indicating the majority of identified hyper-DMRs are reliable. Moreover, even for hyper-DMRs that appeared to be unique to one replicate, the DNA methylation level was also increased in the other replicate, compared to Col-0 control (S4B and S4D Fig), even though the increases in DNA methylation level were not enough to be considered as hyper-DMRs due to our stringent criteria. We then compared the hyper-DMRs between *met18-1* and *met18-2* and obtained 1254 common hyper-DMRs, which account for about 49% and 52% of total hyper-DMRs in *met18-1* and *met18-2*, respectively (S4E and S5 Figs). Similarly, even for one allele-unique hyper-DMRs, DNA methylation level was also increased in the other allele, suggesting that more loci are overlapping between the two alleles than the Venn diagram showed (S4F Fig). Therefore, we used *met18-1* (overlapping hyper-DMRs of *met18-1* two replicates), *met18-2* (overlapping hyper-DMRs of *met18-2* two replicates) and *met18* (overlapping hyper-DMRs of *met18-1* and *met18-2*) hyper-DMRs for subsequent analysis.

### MET18 acts in the same pathway with ROS1 in DNA demethylation

**Fig 4:**
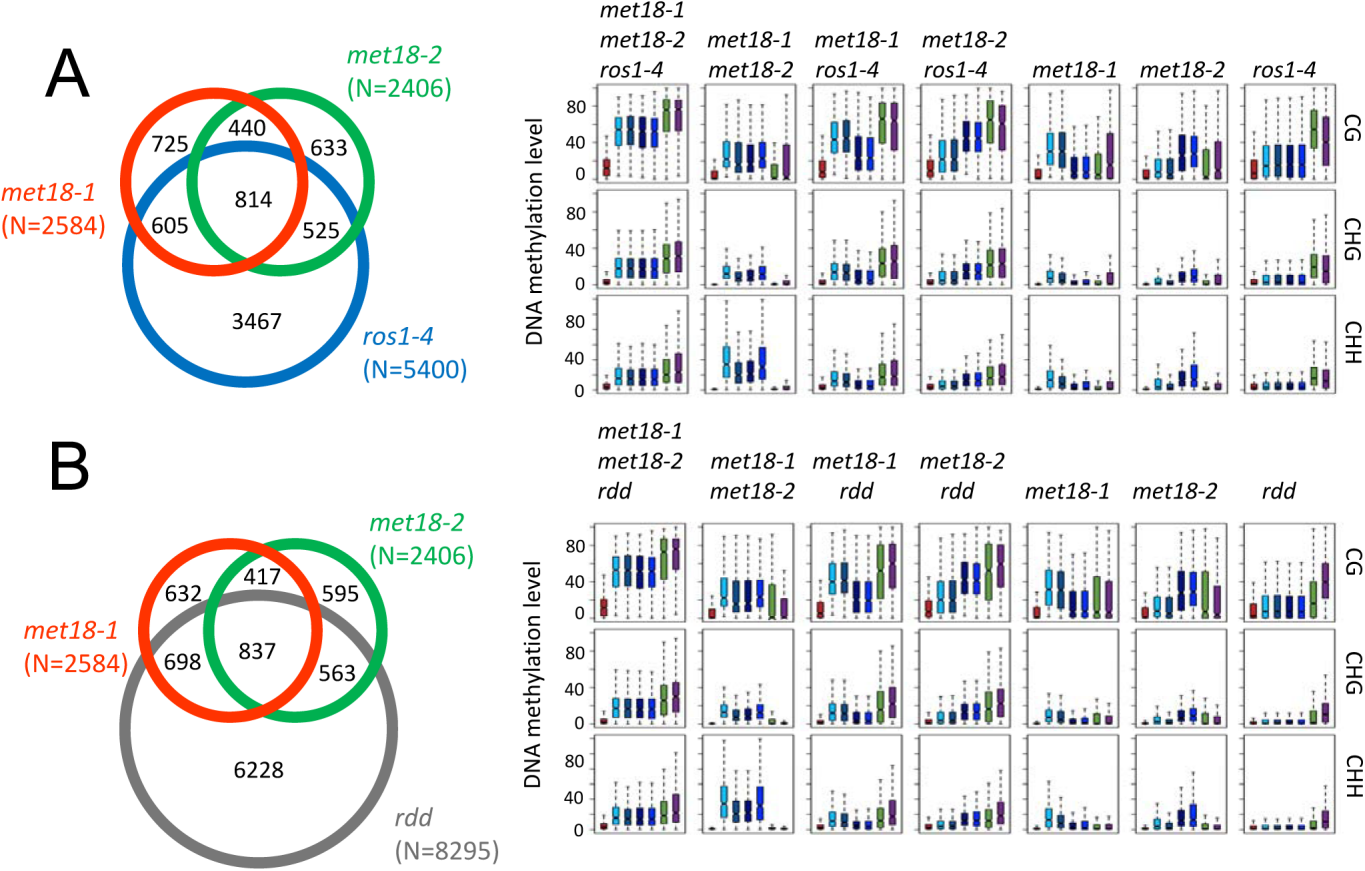
Comparison of hyper-DMRs among *met18-3*, *ros1-4*, and *rdd* mutants. A. Venn diagram showing the numbers of hyper-DMRs that overlap among *met18-1* (Orange), *met18-2* (Green) and *ros1-4* (Blue). Box plots displaying the distribution patterns of average DNA methylation levels (CG, CHG and CHH) calculated from the corresponding overlapping or unique hyper-DMRs. B. Venn diagram showing the numbers of hyper-DMRs that overlap among *met18-1* (Orange), *met18-2* (Green) and *rdd* (Gray). Box plots displaying the distribution patterns of average DNA methylation levels (CG, CHG and CHH) calculated from the corresponding overlapping or unique hyper-DMRs.

ROS1, DML2 and DML3 are the main 5mC DNA glycosylases/demethylases functioning in active DNA demethylation in vegetative tissues in *Arabidopsis* [2,10]. To investigate whether MET18 and ROS1 control a common set of target loci, we compared *met18* DNA methylation data with two published DNA methylomes from the *ROS1* mutant *ros1-4* and a triple mutant of *ROS1* and its two paralogs *DML2*, *DML3*, *rdd* [11,36]. We identified 6151 (5400 hyper- and 751 hypo-DMRs) and 9319 (8295 hyper- and 1024 hypo-DMRs) DMRs in *ros1-4* and *rdd* mutants, respectively (S1 Table and Figs 3A and S5). Based on TAIR10 annotation, we analyzed the distribution of genomic features for the hyper-DMRs in *met18*, *ros1-4* and *rdd* mutants. The results show that the distribution patterns are similar among *met18*, *ros1-4* and *rdd* mutants (Fig 3B and 3C). We then compared the hyper-DMRs of *met18* with those of *ros1-4* and *rdd* mutants. As expected, the majority of the hyper-DMRs in *ros1-4* (64%) overlapped with those in the *rdd* mutant (S6 Fig). For the *met18-1* allele, there are 55% (1419/2584) and 59% (1535/2984) of hyper-DMRs overlapping with those of *ros1-4* and *rdd*, respectively (Fig 4A and 4B, left panels). For the *met18-2* allele, the percentages of overlapping hyper-DMRs with those of *ros1-4* and *rdd* are 56% (1339/2412) and 58% (1400/2412), respectively (Fig 4A and 4B, left panels). For the overlapping hyper-DMRs between *met18-1* and *met18-2* (here refer to as *met18*), about 65% (814/1254) and 67% (837/1254) are overlapping with those of *ros1-4* and *rdd* mutants, respectively (S6 Fig). These results indicate that MET18 and ROS1 control a common set of genomic loci to prevent their DNA hypermethylation. Moreover, the box plots based on hyper-DMR overlap of *met18*-*ros1* and *met18*-*rdd* also indicated that even at *ros1*, *rdd* or *ros1*/*rdd*-unique loci, the average DNA methylation level was higher in *met18* than in Col-0 (Fig 4A and4B, right panels), implying that there are more loci affected by MET18 than the Venn diagram showed (Fig 4A and 4B). Nevertheless, the partial-overlapping patterns also indicate a role of MET18 in controlling ROS1-independent, in addition to ROS1-dependent, DNA demethylation. The heatmaps based on hyper-DMR loci identified in *met18*, *ros1* and *rdd* also suggested that hyper-DMR loci in *met18* were also hypermethylated in *ros1-4* and *rdd* mutants (S7 Fig). Collectively, these results indicate that hyper-DMRs in *met18* highly overlap with those in *ros1-4* and *rdd*, demonstrating that MET18 facilitates DNA demthylation mainly at ROS1-dependent loci.

### The Fe-S motif is critical for ROS1 enzymatic activity

**Fig 5:**
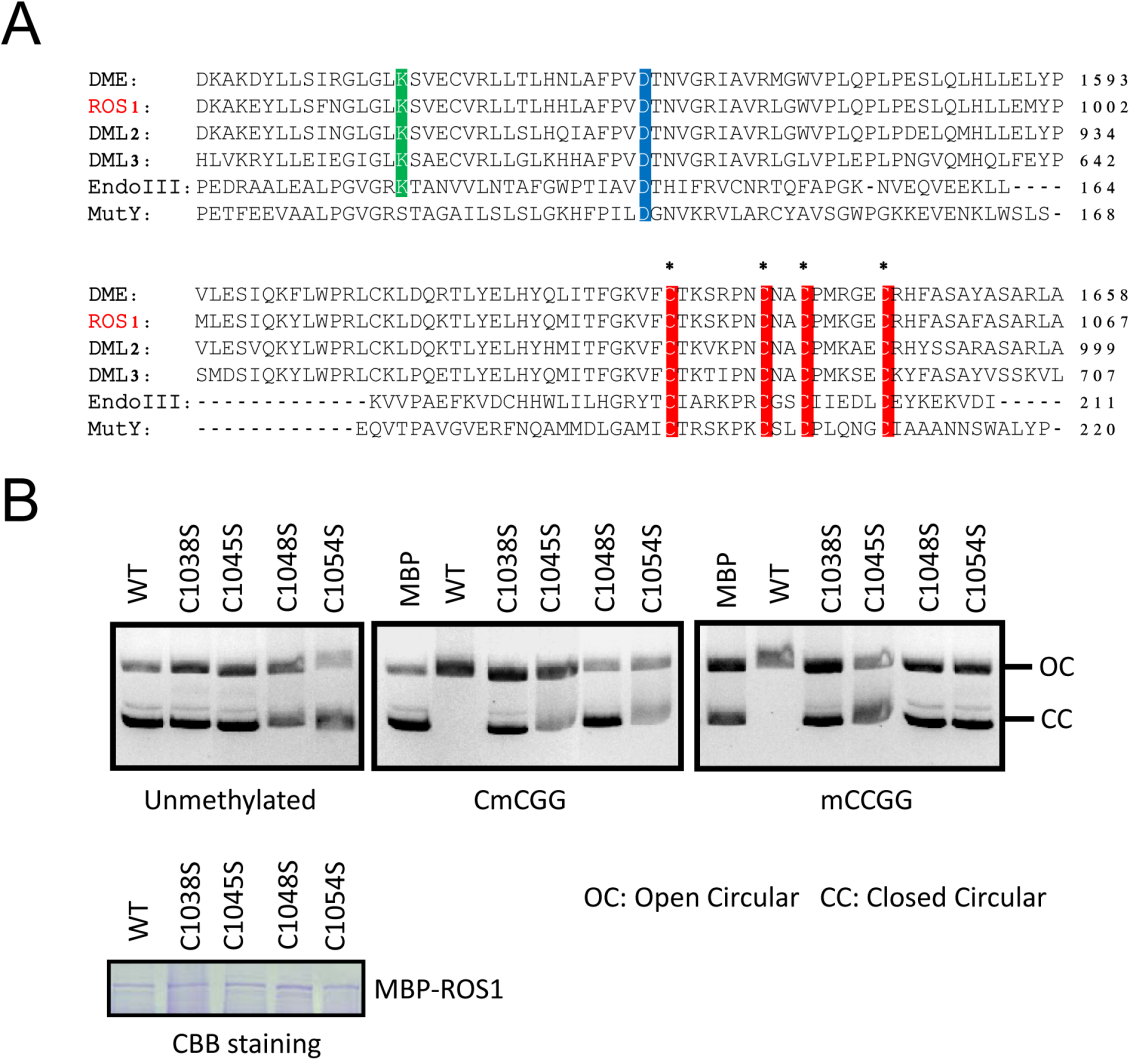
The iron-sulfur-binding motif is required for ROS1 enzymatic activity. A. Protein sequence alignment showing four highly conserved cysteine residues (red) among ROS1 family proteins in *Arabidopsis* and EndoIII and MutY in *E*. *coli*. Two other enzymatically important aspartic acid residues (blue) and lysine residues (green) are also labeled. Asterisks indicate the amino acids that were subjected to site-directed mutagenesis. B. DNA nicking activity assay. Purified recombinant ROS1 and its mutated forms were incubated with purified closed circular (CC) plasmid DNA (methylated and unmethylated). The reaction products were resolved by agarose gel electrophoresis. The quantity of nicks was estimated by the fraction of open circular (OC) plasmids. Unmethylated plasmids were used as a control. Purified MBP protein was used as a negative control to monitor background nuclease activity from *E*. *coli*. Coomassie brilliant blue (CBB)-stained SDS-PAGE gel showing the proper expression of purified wild-type and mutated ROS1 proteins. Equal amount of proteins were loaded.

Proteins in the ROS1 DNA demethylase family contain a conserved Fe-S motif (Fig 5A) which was reported to be required for DME enzymatic activity [22]. To determine whether this motif is important for ROS1 enzymatic activity, we performed a DNA nicking assay as previously described [13]. We generated mutated forms of MBP-ROS1 fusion proteins by changing one of the four conserved cysteine to serine (C to S) and compared their enzymatic activities with that of the wild-type ROS1 protein. The MBP protein was used as a negative control. Coomassie Brilliant Blue (CBB) staining of purified proteins indicated that the mutated forms of ROS1 were properly expressed as wild-type ROS1 (Fig 5B lower panel). We incubated the purified recombinant proteins with either unmethylated or methylated DNA substrates. Any significant DNA nicking activity would result in the disappearance of the fast-migrating supercoiled DNA plasmid. Neither wild-type nor any of the mutated forms of ROS1 could cut the unmethylated substrate. However, wild-type but not the mutated ROS1 could cut the CG- or CHG-methylated substrates (Fig 5B, upper panel). The results show that mutating any of the four conserved cysteines reduces ROS1 activity, suggesting that all four cysteines in the Fe-S-binding motif are critical for ROS1 enzymatic activity (Fig 5B). Consistently, mutations of all 4 Fe-S-binding cysteines in DME were shown to impair DME activity [22].

### The CIA components MET18 and AE7 physically interact with ROS1

**Fig 6:**
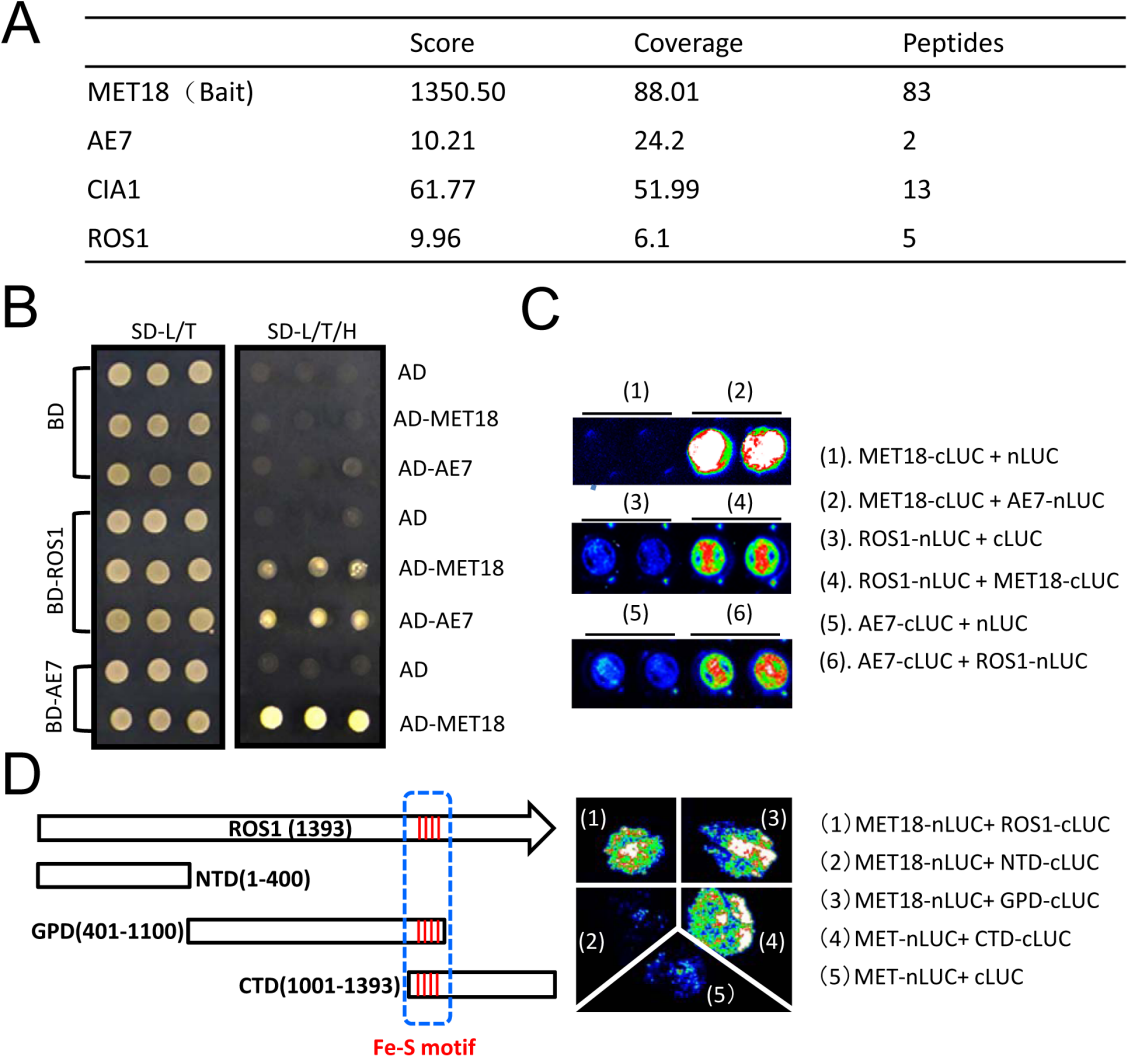
MET18 physically associates with ROS1. A. Mass spectrometric analysis of MET18 affinity purification. Selected unique proteins co-purified from MET18-3FH transgenic plants but not from wild-type are indicated. B.Y2H assays of protein interactions between ROS1, MET18 and AE7. MET18-AE7 interaction served as a positive control. C. Split luciferase assay of interactions between ROS1 and MET18 or AE7 in *Arabidopsis* protoplasts confirmed that ROS1 interacts with MET18 and AE7. ROS1, MET18 and AE7 were transiently expressed in protoplasts by plasmids transfection. D. Split luciferase assay of protein interactions in *tobacco* leaves. The full-length and deletion forms of ROS1 proteins were designated with white boxes. The four conserved amino acids were designated with red line as iron-sulfur motif (blue dashed line). The photograph was taken at 3 day-post-infiltration.

We performed affinity purification of the MET18 complex using inflorescence tissues of Flag-tagged MET18 transgenic plants. The co-purified proteins were identified through mass spectrometric (MS) analyses (Fig 6A and S2 Table). Mass spectrometry showed that AE7 and CIA1, another two components functioning together with MET18 in the CIA pathway, were co-purified with MET18 (Fig 6A and S2 Table). Interestingly, ROS1 was also co-purified from MET18 transgenic plants but not from *35S*::*SUC2* control plant purification. These results suggest that CIA pathway components may interact with ROS1 *in vivo*.

To test for direct interactions between ROS1 and CIA components, yeast two-hybrid (Y2H) assays were performed. Yeast bearing MET18 in the AD vector and AE7 in the BD vector grew well in SD media lacking leucine, tryptophan, or histidine amino acids (SD-L/T/H) (Fig 6B), which is consistent with a previous report that MET18 directly interacts with AE7 [27]. The direct interaction of AE7 and MET18 was further confirmed by split luciferase assay in *Arabidopsis* protoplasts (Fig 6C). Yeast bearing ROS1 in the BD vector and MET18 in AD the vector also grew in the SD-L/T/H media, demonstrating that MET18 directly interacts with ROS1. Yeast bearing AE7 in the AD vector and ROS1 in the BD vector also grew in the SD-L/T/H media (Fig 6C), suggesting AE7 can also directly bind to ROS1. Moreover, the interactions of ROS1 with MET18 and AE7 were further confirmed by a split luciferase assay (Fig 6C). These results demonstrate that ROS1 physically interacts with the CIA components MET18 and AE7. To gain insights into the interaction domain of ROS1 with MET18, we generated truncated forms of ROS1, including the N terminal lysine-rich domain (NTD), the HhH-GPD motif-containing GPD domain and the C terminal domain (CTD)[37] (Fig 6D). The iron-sulfur motif is located in the overlapping region (100 amino acids) of GPD and CTD domain (Fig 6D). Split luciferase assay was performed to test the interactions between MET18 and the different forms of ROS1. The results showed that, besides full length ROS1, MET18 can also interact with ROS1GPD and ROS1CTD, but not with ROS1NTD (Fig 6D). This result suggests that the iron-sulfur motif may be the critical region for MET18-ROS1 interaction.

## Discussion

DNA methylation and histone modifications are major epigenetic marks determining transcriptional activation or silencing of the associated genes [1,2]. In *Arabidopsis*, DNA demethylation mediated by the 5mC DNA glycosylase ROS1 prevents thousands of genomic loci from being hyper-methylated [11] and prevents transcriptional silencing at some loci [6,10,38,39]. In our previous searches for anti-silencing factors in a genetic screen, we identified many *ros1* mutant alleles, illustrating that ROS1 is required to prevent the silencing of the *35S*::*SUC2* transgene [32–34]. Using the same system, we identified the *asi3-1* mutant and mapped the mutation to the *MET18* locus. Whole-genome bisulfite sequencing identified thousands of hyper-methylated loci in *met18* mutants (Fig 3 and S1 Table), indicating that MET18 is a new factor controlling DNA demethylation. The comparison of DNA methylomes illustrated that hyper-DMRs in the *met18* mutant highly overlap with those identified in *ros1-4* and *rdd* mutants (Fig 4), supporting that MET18 functions in the ROS1/DML2/DML3-mediated DNA demethylation.

AtMET18 is a homolog of the yeast MET18 protein, which was identified as a component of the cytoplasmic Fe-S assembly machinery [27]. In *Arabidopsis*, AtMET18 forms a protein complex with other CIA components including AE7, CIA1, and NAR [27]. About 25 years ago, a DNA glycosylase functioning in base excision repair was found to contain an Fe-S cluster that was essential for its activity [40]. A recent study showed that mutation in the conserved Fe-S motif of DEMETER, a ROS1 paralogue in *Arabidopsis*, impaired the protein’s enzymatic activity in base excision repair [22]. Our site-directed mutagenesis analysis of ROS1 in a nicking assay showed that the iron-sulfur motif is also critical for ROS1 enzymatic activity (Fig 5). This result suggests that the enzymatic activity of ROS1 may require MET18-mediated Fe-S assembly. Interestingly, ROS1 was detected with other CIA components in the MET18-containing protein complex (Fig 6A). Our Y2H and split luciferase assays further confirmed that MET18 directly interacts with ROS1 and AE7 (Fig 6B and 6C). AE7 also directly interacted with ROS1. Thus, MET18 and AE7 may have partially redundant functions in delivering Fe-S clusters to target proteins. It is unclear how CIA components select target apoproteins because no conserved amino acid sequence was identified in the Fe-S proteins [41]. Our results are consistent with previous reports that CIA components target specific apoproteins via physical interaction [31,42]. Therefore, it is important that future studies determine the structural features that enable recognition between CIA components and the target apoproteins.

Biogenesis of Fe-S clusters is a multistep process that takes place in mitochondria and the cytoplasm, but how it is linked to nuclear Fe-S proteins is not known [30]. ROS1-mediated DNA demethylation is a nuclear process [10]. Our localization assay in tobacco leaves indicated that MET18 is mainly localized to the cytoplasm, although occasional localization in the nucleus was also observed (S8 Fig). ROS1 functions in the nucleus [10]. Luo et al. (2012) reported that the MET18-C terminus interacts with AE7 in both the nucleus and cytoplasm in bimolecular fluorescence complementation (BiFC) assays [27]. It is possible that the CIA machinery may facilitate ROS1 protein maturation in the cytoplasm before ROS1 is translocated into the nucleus.

Based on our results, a working model for the anti-silencing and DNA demethylation roles of MET18 can be proposed (S9 Fig). In this model, MET18 and other CIA components transfer an Fe-S cluster onto one of their apoproteins, ROS1, in the nucleus or cytoplasm. The mature ROS1 protein is then enzymatically active and recruited to the *35S*::*SUC2* promoter or other methylated genomic loci to remove methylated cytosines. The promoter is then demethylated, allowing for active transcription of the *SUC2* transgene. The model helps to explain an earlier observation that a defect in AE7 causes DNA hypermethylation at two loci known to be ROS1 targets [27].

CIA proteins are important for the replication of DNA and the maintenance of genomic integrity [20,24,31,43–45]. Consistent with the importance of the CIA pathway in plants, both EMS and T-DNA insertion mutants of MET18 displayed a stunted phenotype (S1 Fig), indicating that MET18 is also indispensable for normal development. Unlike AE7, NAR1, and CIA1, whose homozygous mutants are lethal, the *met18* mutant can grow and propagate normally, indicating that MET18 is less important than the other CIA components and there may be other proteins that are functionally redundant with MET18. Although there are no other MET18 homologs in Arabidopsis, other proteins in the CIA pathway such as AE7 may also be capable of delivering the Fe-S cluster to some apoproteins. This notion is consistent with our finding that not only MET18 but also AE7 could interact with ROS1 (Fig 6). Therefore, it can be speculated that, without MET18, some ROS1 protein probably can still retain Fe-S clusters and confer demethylation activity at some loci. This hypothesis can explain the observation that in some of the hyper-DMR loci *met18* hypermethylation is not as strong as in the *ros1* mutant.

The *met18ros1* double mutant displayed the same phenotype of reduced size as the *met18* single mutant but the *ros1* single mutant or the *rdd* mutant displayed a normal growth phenotype (S1 Fig), indicating that MET18 participates in additional processes besides the ROS1-mediated DNA demethylation. Collectively, our study reveals that MET18 affects transgene silencing and DNA methylation by interacting with the DNA demethylase ROS1 for Fe-S delivery. Our study thereby demonstrates a direct linkage between the CIA pathway and ROS1-mediated active DNA demethylation. Through metabolites such as acetyl-CoA, SAM and SAH, which are important in histone and DNA modifications, the state of cellular metabolism is intimately connected to epigenetic modifications [46]. Knowledge of such connections is essential for our understanding of epigenetic regulation and how nutrition and metabolism affect development and diseases through epigenetic regulation [47]. In this regard, the linkage between the CIA pathway and active DNA demethylation represents an important but previously underappreciated connection between epigenetics and nutrition and metabolism.

## Materials and Methods

### Plant materials and growth conditions

All plants were grown under a long-day photoperiod (16-h light/8-h dark). 1/2-strength Murashige and Skoog (MS) medium containing 1% sucrose was used for studying root phenotype. All other experiments used growth medium with 1% glucose. Kanamycin of 100 mg/L was used for the kanamycin-sensitivity assay. Seedlings were photographed 14 days after germination.

Two other alleles of the *met18* mutant, *met18-1* (SALK_121963) and *met18-2* (SALK_147068), were obtained from the Arabidopsis Biological Resource Center (ABRC) and confirmed by PCR-based genotyping [35]. *MET18* genomic DNA including the upstream 2-kb promoter region was amplified by PCR and cloned into a modified pEarleyGate 302 vector [48], which bears three copies of FLAG and HA tags. The construct was introduced into the met18-3 mutant by *Agrobacterium*-mediated transformation via the floral dip method [49] All primers used in the study are listed in S3 Table.

### Mutant screening and map-based cloning

The *met18-3* mutant was obtained by a screen method described in our previous report [33,34]. In brief, EMS-treated M2 seeds were grown on ½ MS medium containing 1% sucrose. Seedlings with roots longer than the wild type plants were selected as potential transgene silencing mutants. To clone the *ASI3* gene, the *asi3-1* mutant was crossed with Landsberg erecta, and F1 plants were self-pollinated to obtain the F2 population. F2 seeds were planted on 1/2 MS medium containing 1% sucrose and 25 μg/mL hygromycin. Seedlings with normal, long roots were selected for genetic mapping in order to calculate *asi3-1* linkage. The *asi3-1* mutant genomic DNA was then re-sequenced to determine the location of the mutation in the mapping region.

### Analysis of DNA methylation levels

DNA methylation-sensitive PCR was performed according to a previous report [34]. In brief, genomic DNA was extracted from 2-week-old seedlings using the DNeasy Plant Minikit (QIAGEN) and was quantified with NanoDrop 2000c UV-Vis Spectrophotometer (Thermo SCIENTIFIC). About 50 ng of DNA was subjected to DNA methylation-sensitive restriction enzyme digestion in a 50-μL reaction. The digested DNA was used as template for PCR, and the PCR products were subjected to agarose gel electrophoresis. The non-digested genomic DNA was used as control template for PCR.

For whole-genome bisulfite sequencing, DNA was extracted from 2-week-old seedlings. The DNA samples were submitted to BGI (Shenzhen, China) for bisulfite treatment and DNA sequencing. The bisulfite treatment and DNA methylation analysis were performed as previously described [50]. When counting the number of overlapping DMRs, if aregion in one mutant overlaps with several regions in another mutant, we calculated the number of overlapping regions from the perspective of *met18*, *ros1-4*, and *rdd*. For TE annotation, if a region overlaps with a TE or a transposable elemental gene, the region is classified as a TE region. Regions having no overlap with TE or transposable element gene and overlapping with protein-coding genes are classified as Gene regions.

### Real-time quantitative RT-PCR

For real-time quantitative RT-PCR, total RNAs were extracted from 2-week-old seedlings using the RNeasy Plant Minikit (QIAGEN). After TURBO DNase I treatment (Ambion), 2 μg of RNA was subjected to RT reaction using the SuperScript III First-Strand Kit according to the manufacturer’s instructions (Invitrogen). The 1st-strand cDNAs were then amplified using IQ SYBR green supermix (BIO-RAD) with the CFX96 real-time PCR detection system (BIO-RAD).

### ROS1 enzymatic activity assay

The MBP-ROS1 in the pMAL-c2x vector was constructed as previously reported [6,51]. Site-directed mutagenesis was performed via PCR to obtain the expression constructs of ROS1C1038S-, ROS1C1045S-, ROS1C1048S-, and ROS1C1054S-mutant proteins. All constructs were transformed into *dcm* ^-^ Codon Plus cells of *E*. *coli* strain BL21. The induction and purification of ROS1 and mutant proteins were performed as previously described [15]. The ROS1 enzymatic activity assay (the nicking assay) was performed as previously described [13]. In brief, plasmid pBluescript KS purified from the *dcm-* strain of *E*. *coli* BL21 (DE3) was methylated *in vitro* with MspI (mCHG) or SssI (mCG) methylases (NEB). The methylation status was confirmed by digestion with MspI and HpaII restriction endonucleases. The unmethylated plasmid was used as a control. The purified, closed-circular plasmids (250 ng) were then incubated with purified MBP-ROS1 protein or its mutated forms in nicking buffer containing 40 mM Hepes-KOH (pH 8.0), 0.1 M KCl, 0.5 mM EDTA, 0.5 mM DTT, and 0.2 mg/ml BSA at 37°C for 2 h. After adding stop solution (0.4 M EDTA and 1% SDS), the reaction mixtures were heated at 70°C for 5 min and subjected to 1% agarose gel electrophoresis.

### Yeast two hybrid (Y2H), split luciferase assays and cellular localization

The full-length coding regions of *MET18*, *ROS1*, and *AE7* were first individually cloned into the pENTR/D-TOPO directional cloning vector (Invitrogen) and then transferred into the destination vectors pDEST 22 (AD) and pDEST 32 (BD) (Invitrogen) via LR recombination using Gateway Clonase II Enzyme (Invitrogen).

For the split luciferase assay, constructs carrying full-length coding regions of *MET18*, *AE7*, and full-length/truncated forms of *ROS1* fused with split luciferase in the pEarleyGate vector were co-transfected into Arabidopsis protoplast or *Nicotiana Benthamiana* for overnight incubation or 3-day-growth, respectively [48]. The empty vectors was used as a negative control. The luciferase activity was determined using CCD camera equipped with Winview software (Princeton instruments). Protoplast was prepared as previously reported [52]. For MET18-GFP cellular localization, Agrobacterium containing MET18-GFP construct driven by 35S promoter was infiltrated into *Nicotiana benthamiana* leaves. The cellular localization was examined by Zeiss Microscope at 3 day-post-infiltration.

### Affinity purification and mass spectrometry

For affinity purification of MET18 and its associated proteins, 5 g of flower tissues were collected from *MET18-3FLAG-3HA* transgenic plants, and tissue from *35S*::*SUC2* plants was used as a negative control. Extraction of total proteins and affinity purification were performed as described previously [53] with minor modifications. Briefly, flower tissues were ground to fine powders in liquid nitrogen and suspended in 30 ml of lysis buffer (50 mM Tris pH 7.6, 150 mM NaCL, 5 mM MgCl_2_, 10% glycerol, 0.1% NP-40, 0.5 mM DTT, 1 mM PMSF, and 1 protease inhibitor cocktail tablet (Roche)). After further homogenization by douncing and centrifugation at 4°C, the supernatants were incubated with 120 μL of anti-FLAG M2 agarose beads (Sigma), which had been pre-equilibrated with lysis buffer. After incubation at 4°C with rotation for 2–3 hours, the agarose beads were washed three times for 5 minutes each time with 40 ml of lysis buffer, three times for 5 minutes each time with 1 mL of lysis buffer, and three times for 5 minutes each time with 1 ml of PBS buffer. The agarose beads were finally resuspended in 120 μL of PBS buffer for mass spectrometry according to a previous report [54].

### Accession numbers

The raw WGBS sequencing dataset of met18-1 rep.1, met18-1 rep.2, met18-2 rep.1 and met18-2 rep.2 was deposited to the public database NCBI GEO (accession number GSE69281). The Col-0, *ros1-4* and *rdd* whole genome bisulfite sequencing data were from GEO accession GSE33071 [11].

## Supporting Information

S1 Fig Developmental phenotypes of *met18* and *met18ros1* double mutants and identification of complementation lines. (A) Morphology phenotypes of *35S*::*SUC2*, *met18-3* and complementation plants. *met18-3* mutant plants are smaller than *35S*::*SUC2* plants, and *MET18* complementation rescues the developmental defects. Seedlings were photographed 40 days after germination. (B) Western blot of T1 MET18-3FH transgenic lines. Three representative lines of MET18-3FH transgenic plants in the T1 generation were selected for detecting the recombinant protein. Total proteins were extracted from leaves, and anti-FLAG antibody was used to detect the recombinant protein. *35S*::*SUC2* plants were used as a negative control. CBB-stained SDS-PAGE gel served as the loading control. (C and D) Developmental phenotypes of *met18 and ros1* single mutants as well as their double mutant in Col-0 (C) and *35S*::*SUC2* background (D). Like the *met18* mutants, the double mutants were smaller than wild-type plants. In contrast, the *ros1* mutants were normal in size.
(PDF)

S2 Fig *ROS1* transcript levels in *35S*::*SUC2* and *met18-3* mutant. Quantitative RT-PCR was performed to compare *ROS1* expression in *35S*::*SUC2* and *met18-3* seedlings. *ACTIN2* was used as the internal control.
(PDF)

S3 Fig Summary of whole genome bisulfite sequencing technical parameter generated in this study. (PDF)

S4 Fig Overlaps of hyper-DMRs and distributions of average DNA methylation levels in corresponding regions. Venn diagrams showing hyper-DMRs overlap between two biological replicates of the same *met18* allele (A, C) and between two different *met18* alleles (E). Box plots (B, D and F) showing the distributions of average cytosine methylation levels (CG, CHG and CHH) calculated from the overlapping or unique hyper-DMRs in A, C and E, respectively.
(PDF)

S5 Fig The distributions of hyper-DMR loci of *met18-3*, *ros1-4*, and *rdd* mutants along five chromosomes. Chr. Chromosome.
(PDF)

S6 Fig The Venn diagram showing the numbers of hyper-DMRs that overlap among *met18* (Purple), *ros1-4* (Blue) and *rdd* (Gray). Here, met18 represents the overlapping DMRs of all four met18 methylomes (*met18-1* rep.1, *met18-1* rep.2, *met18-2* rep.1 and *met18-2* rep.2)
(PDF)

S7 Fig Heat maps of DNA methylation levels in different cytosine contexts (CG, CHG, and CHH) in *met18*, *ros1-4*, and *rdd* mutants. DNA methylation levels in different cytosine contexts were calculated at hyper-DMR loci in *met18-1*, *met18-2*, *ros1-4* and *rdd* mutants. Methylation levels are represented by colors indicating low and high methylation levels.
(PDF)

S8 Fig Cellular localization of MET18 in tobacco leaves. The cellular and subcellular localizations of MET18 were detected by expressing *35S-MET18-GFP* and *35S-ROS1-GFP* in *N*. *benthamiana* leaves. Photographs were taken at 3 day-post-infiltration.
(PDF)

S9 Fig A working model of anti-silencing and DNA demethylation mediated by MET18. In the absence of MET18, ROS1 protein has low enzymatic activity and is unstable because it lacks the Fe-S complex; this results in hypermethylation in the 35S promoter region and transcriptional silencing of the *SUC2* transgene. In the presence of MET18, the MET18-associated CIA complex, including CIA1, NAR1, and AE7, transfers the Fe-S cluster onto the ROS1 protein via direct interactions between ROS1 and MET18/AE7. Activative ROS1 is then able to carry out DNA demethylation reactions at the 35S promoter for DNA demethylation. As a result, expression of *SUC2* transgene is released from DNA hypermethylation.
(PDF)

S1 Table Hyper-DMRs identified in *met18*, *ros1-4*, and *rdd* mutants. (XLSX)

S2 Table A list of proteins identified by MET18 affinity purification followed by mass spectrometry. (XLSX)

S3 Table Primers used in this study. (XLSX)
