# Supplementary figures and images for: MET18 Connects the Cytosolic Iron-Sulfur Cluster Assembly Pathway to Active DNA Demethylation in Arabidopsis
Source: PLoS Genet. 2015 Oct 22;11(10):e1005559. doi: 10.1371/journal.pgen.1005559 (PMC4619598; doi:10.1371/journal.pgen.1005559)

# Figure S1

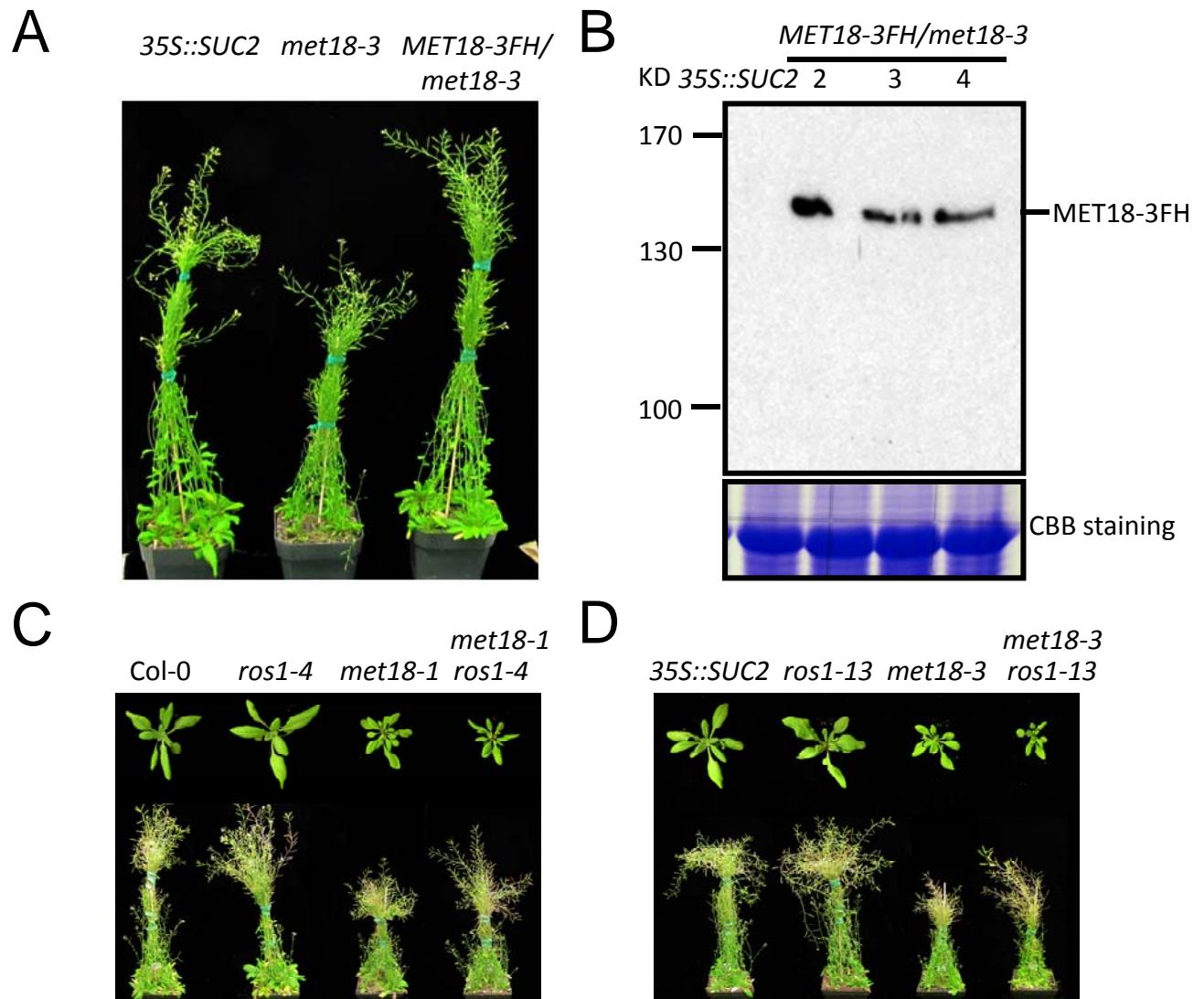

Supplement: S1 Fig — (A) Morphology phenotypes of 35S::SUC2, met18-3 and complementation plants. met18-3 mutant plants are smaller than 35S::SUC2 plants, and MET18 complementation rescues the developmental defects. Seedlings were photographed 40 days after germination. (B) Western blot of T1 MET18-3FH transgenic lines. Three representative lines of MET18-3FH transgenic plants in the T1 generation were selected for detecting the recombinant protein. Total proteins were extracted from leaves, and anti-FLAG antibody was used to detect the recombinant protein. 35S::SUC2 plants were used as a negative control. CBB-stained SDS-PAGE gel served as the loading control. (C and D) Developmental phenotypes of met18 and ros1 single mutants as well as their double mutant in Col-0 (C) and 35S::SUC2 background (D). Like the met18 mutants, the double mutants were smaller than wild-type plants. In contrast, the ros1 mutants were normal in size. (PDF) [file pgen.1005559.s001.pdf]

# Figure S2

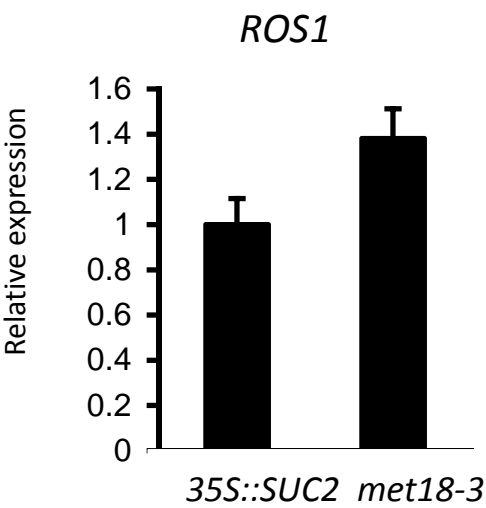

Supplement: S2 Fig — Quantitative RT-PCR was performed to compare ROS1 expression in 35S::SUC2 and met18-3 seedlings. ACTIN2 was used as the internal control. (PDF) [file pgen.1005559.s002.pdf]

# Figure S4

**A**

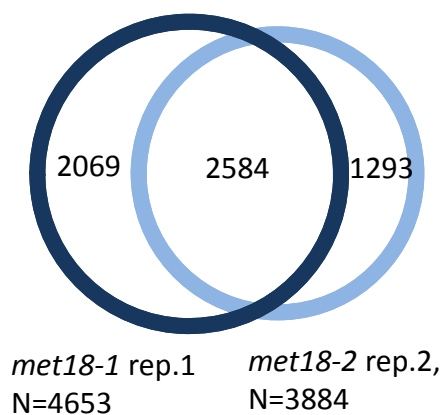

**B**

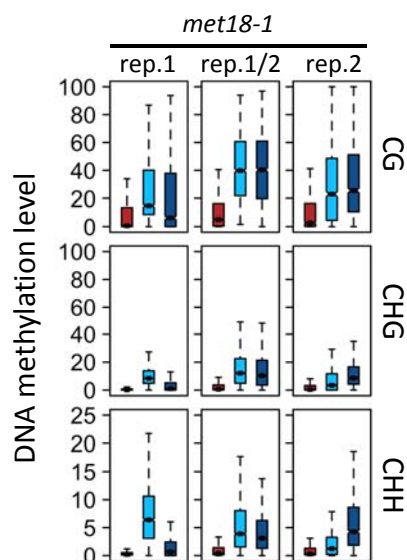

**C**

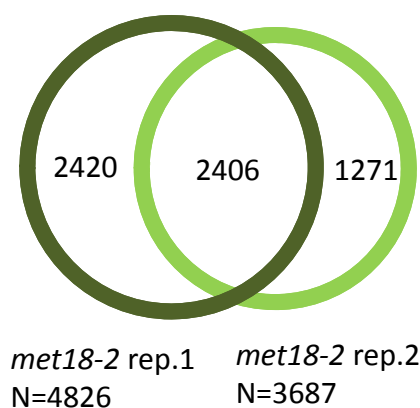

**D**

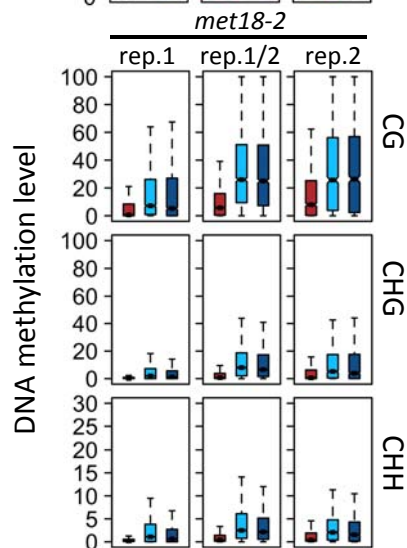

**E**

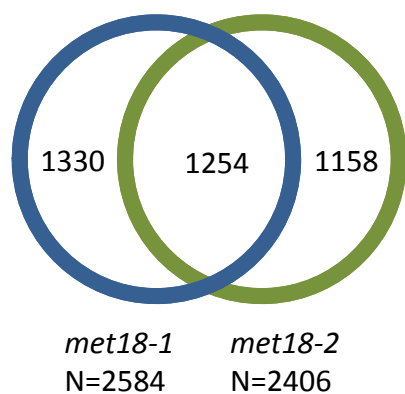

**F**

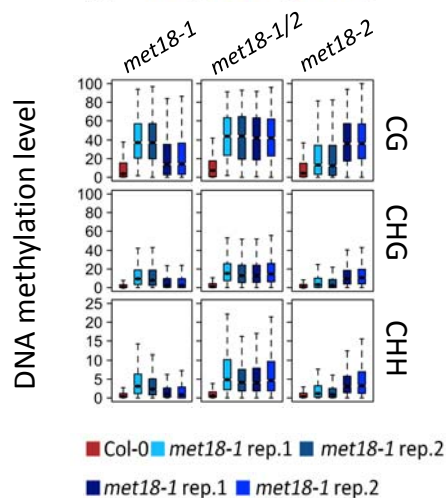

Supplement: S4 Fig — Venn diagrams showing hyper-DMRs overlap between two biological replicates of the same met18 allele (A, C) and between two different met18 alleles (E). Box plots (B, D and F) showing the distributions of average cytosine methylation levels (CG, CHG and CHH) calculated from the overlapping or unique hyper-DMRs in A, C and E, respectively. (PDF) [file pgen.1005559.s004.pdf]

Figure S5

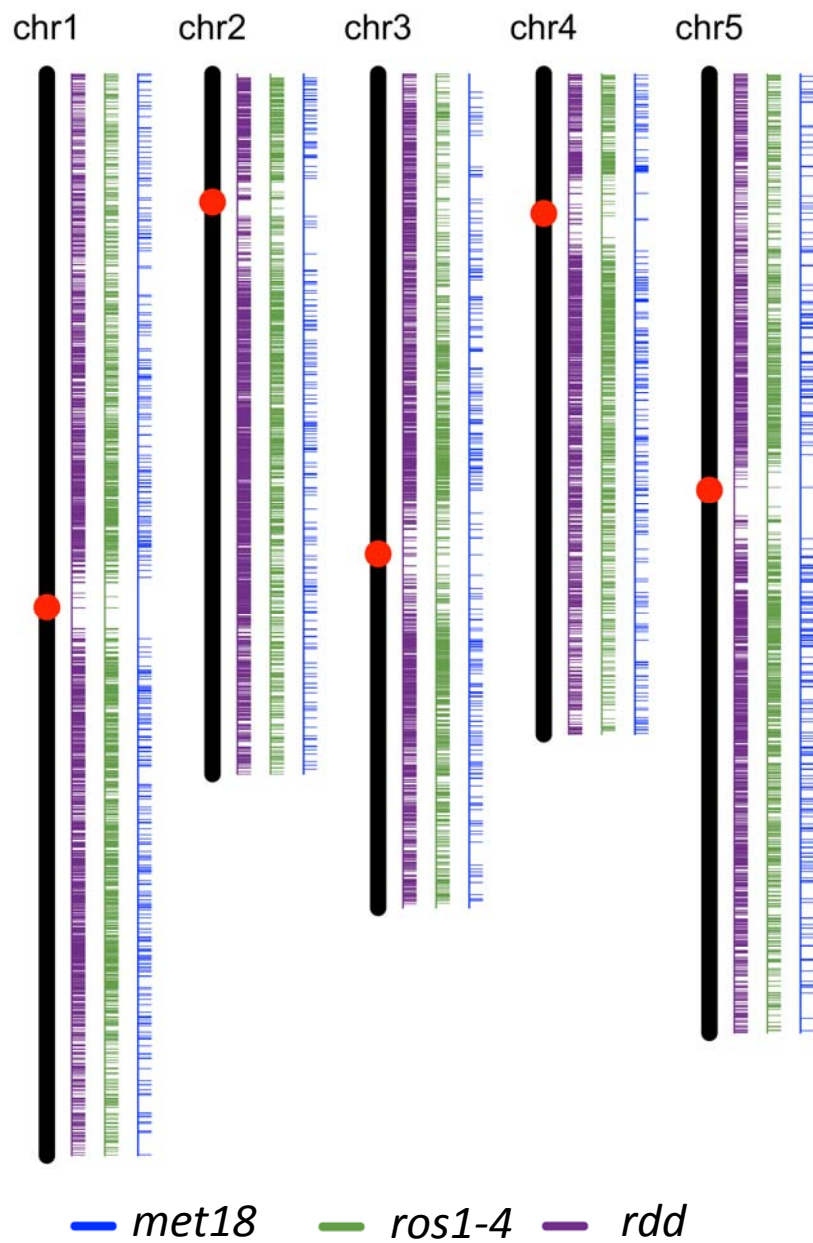

Supplement: S5 Fig — Chr. Chromosome. (PDF) [file pgen.1005559.s005.pdf]

# Figure S6

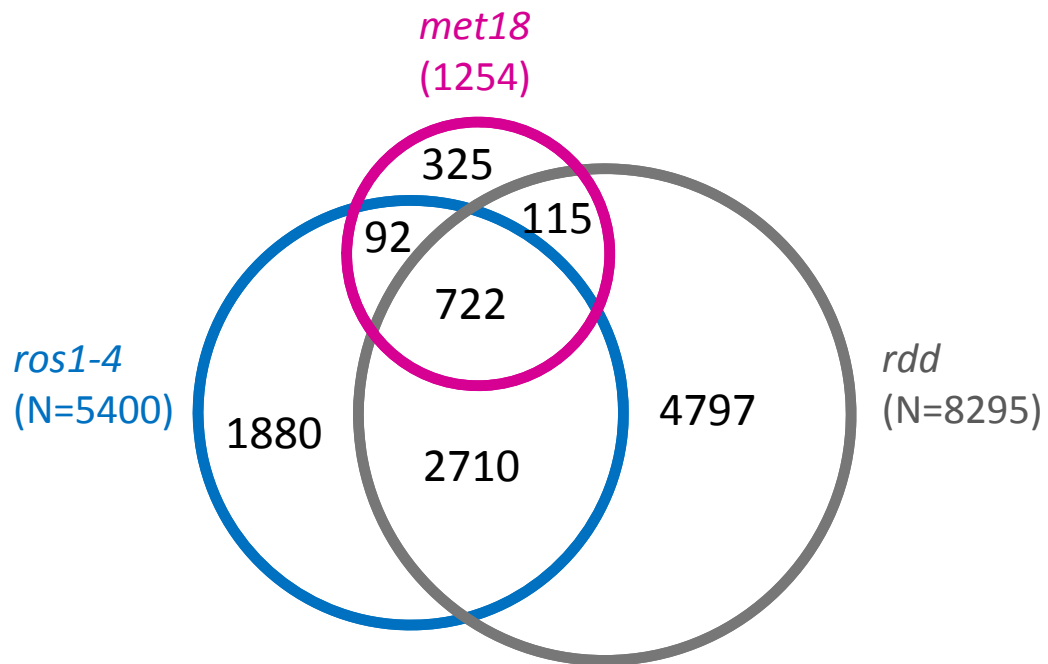

Supplement: S6 Fig — Here, met18 represents the overlapping DMRs of all four met18 methylomes (met18-1 rep.1, met18-1 rep.2, met18-2 rep.1 and met18-2 rep.2) (PDF) [file pgen.1005559.s006.pdf]

Figure S7

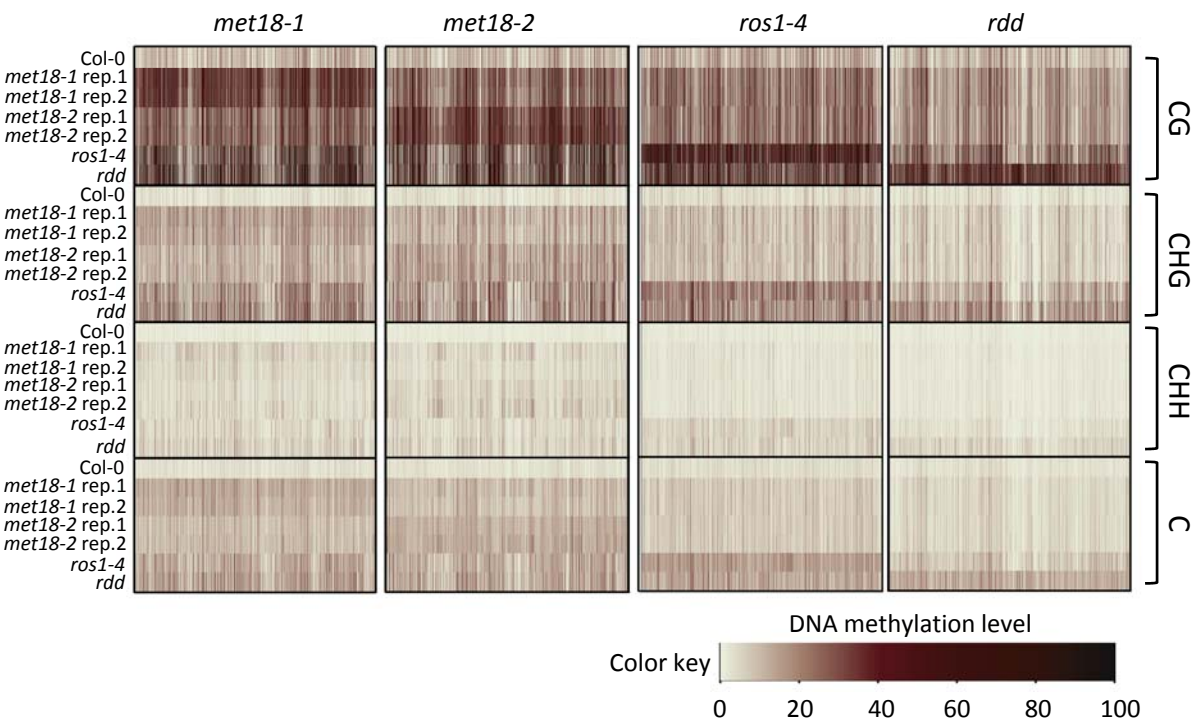

Supplement: S7 Fig — DNA methylation levels in different cytosine contexts were calculated at hyper-DMR loci in met18-1, met18-2, ros1-4 and rdd mutants. Methylation levels are represented by colors indicating low and high methylation levels. (PDF) [file pgen.1005559.s007.pdf]

# Figure S8

MET18-GFP

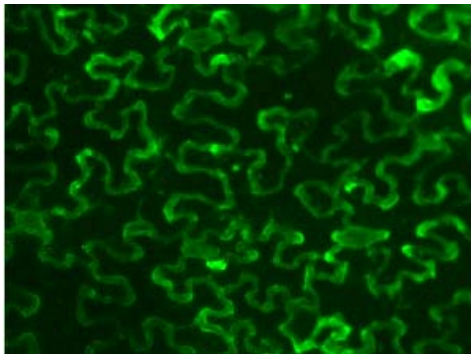

Merge

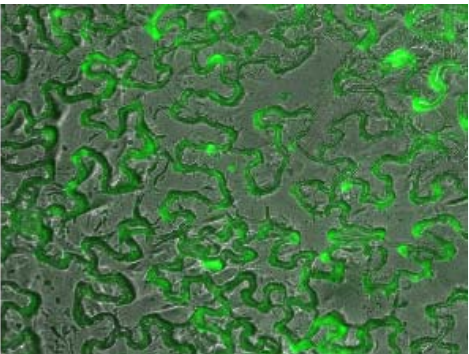

Supplement: S8 Fig — The cellular and subcellular localizations of MET18 were detected by expressing 35S-MET18-GFP and 35S-ROS1-GFP in N. benthamiana leaves. Photographs were taken at 3 day-post-infiltration. (PDF) [file pgen.1005559.s008.pdf]

# Figure S9

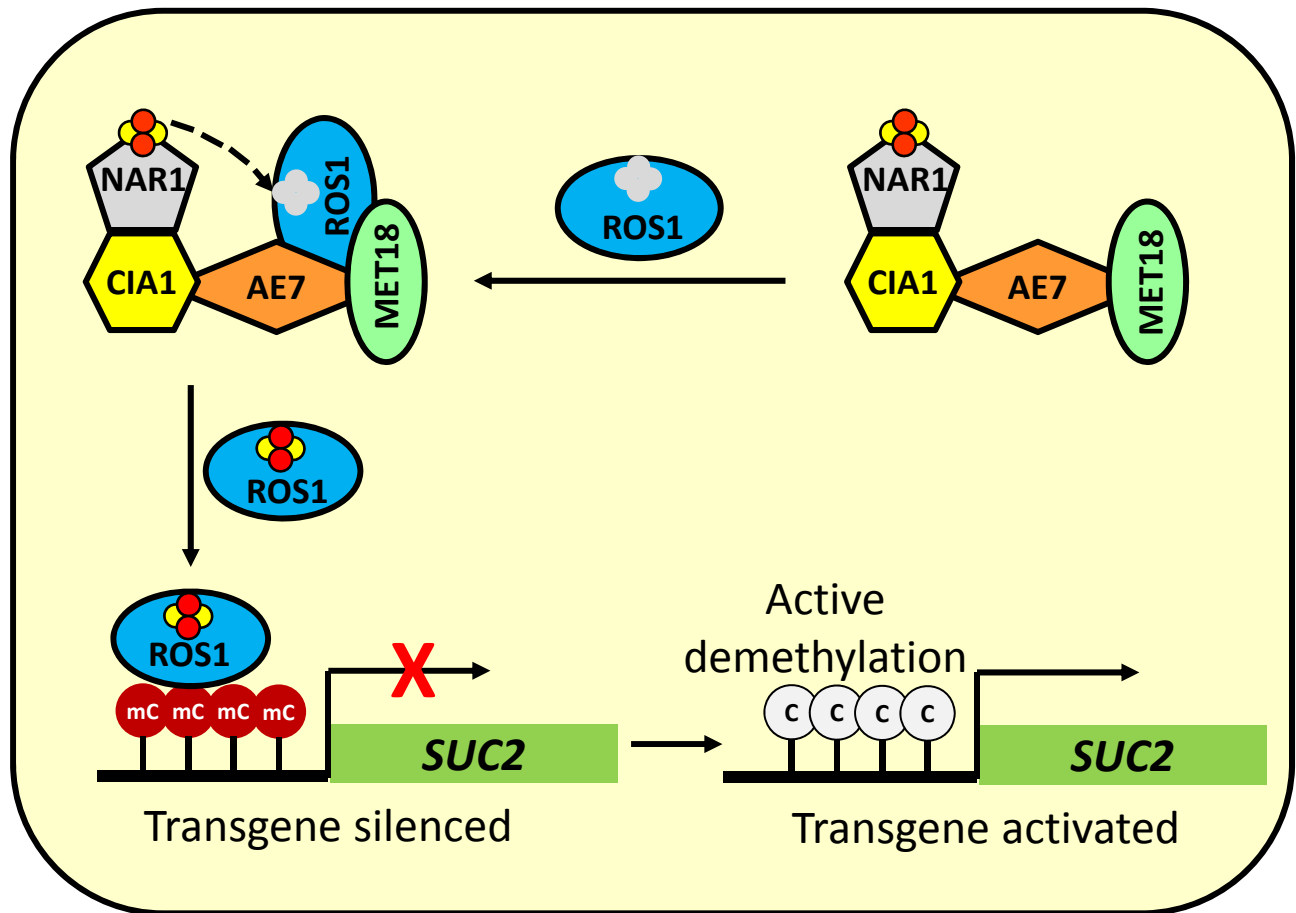

Supplement: S9 Fig — In the absence of MET18, ROS1 protein has low enzymatic activity and is unstable because it lacks the Fe-S complex; this results in hypermethylation in the 35S promoter region and transcriptional silencing of the SUC2 transgene. In the presence of MET18, the MET18-associated CIA complex, including CIA1, NAR1, and AE7, transfers the Fe-S cluster onto the ROS1 protein via direct interactions between ROS1 and MET18/AE7. Activative ROS1 is then able to carry out DNA demethylation reactions at the 35S promoter for DNA demethylation. As a result, expression of SUC2 transgene is released from DNA hypermethylation. (PDF) [file pgen.1005559.s009.pdf]
