# Supplementary material for: MET18 Connects the Cytosolic Iron-Sulfur Cluster Assembly Pathway to Active DNA Demethylation in Arabidopsis
Source: PLoS Genet. 2015 Oct 22;11(10):e1005559. doi: 10.1371/journal.pgen.1005559 (PMC4619598; doi:10.1371/journal.pgen.1005559)
Supplement: S3 Fig — (PDF) [file pgen.1005559.s003.pdf]

Figure S3

Sample	Raw Reads	Uniquely Mapped Reads	reads after remove duplicate	average coverage per C	conversion rate
met18-1 rep.1	64389274	45656340	30393943	11.437	99.92%
met18-1 rep.2	70426848	51560399	33743130	12.669	99.93%
met18-2 rep.1	84342152	64120473	43675810	16.837	99.92%
met18-2 rep.2	36243520	27484224	19844373	7.346	99.97%
